# Going north: adaptation of soybean to long-day environments

**DOI:** 10.1093/jxb/erad105

**Published:** 2023-05-19

**Authors:** Johann Vollmann, Mária Škrabišová

**Affiliations:** Department of Crop Sciences, University of Natural Resources and Life Sciences Vienna, 3430 Tulln an der Donau, Austria; Department of Biochemistry, Faculty of Science, Palacký University in Olomouc, 78371 Olomouc, Czech Republic

**Keywords:** Flowering time regulation, maturity, photoperiod, soybean, stem termination

## Abstract

This article comments on:

**Zhu X, Leiser WL, Hahn V, Würschum T.** 2023. The genetic architecture of soybean photothermal adaptation to high latitudes. Journal of Experimental Botany **74,**2987–3002

**In plant breeding, understanding genetic variation in the photoperiodic control of flowering time of crop plants such as soybean is a prerequisite for managing adaptation to new environments. Zhu *et al*. (2023) analyzed a large diversity panel of >1500 early maturity soybean lines to disclose the genetic architecture behind the timing of flowering and maturity. Their findings confirm known maturity loci and reveal new candidate genes and alleles as well as environmental interactions of individual quantitative trait loci (QTLs) for flowering and maturity time. The results shed light on the complexity of the regulatory network which controls the timing of flowering in soybean. This supports the fine-tuning of plant architectures through the combination of stem termination and flowering genes towards a better adaptation of soybean to high latitudes or other stressful environments.**

In high latitude environments, proper timing of the reproductive cycle is the most important feature of environmental adaptation in annual flowering plants to reach maturity and viable seed development before killing frost occurs. Photoperiod and vernalization have been identified as the most relevant pathways to control flowering time in addition to different endogenous factors including gibberellic acid, plant age, and other regulators. With respect to photoperiod, day length is perceived by photoreceptors in leaves, and long-distance signals such as the *Arapidopsis thaliana* regulator *FLOWERING LOCUS T* (*FT*) can initiate the flowering process at the shoot apex or in axillary meristems (Srikanth and Schmid, 2011). In agricultural crop plants, the appropriate regulation of flowering time is a prerequisite for the extension of planting areas away from the original place of domestication to regions diverging in latitude. Therefore, breeding for adaptation to new target environments differing in latitude and day length requires genetic variation in response to photoperiod, which is particularly relevant for optimizing the yield potential of short-day plants such as maize or soybean when transferred to high latitude regions.

Soybean (*Glycine max* [L.] Merr.) is an important source of protein and oil for human food and animal feed production grown annually on >125 Mha world-wide (FAOSTAT, 2023). The rapid expansion of soybean acreage in the tropics for livestock feed production is associated with massive deforestation, increased greenhouse gas emission, and loss of biodiversity (Song *et al*., 2021). Alternatively, soybean production in higher latitude arable farming regions through sustainable intensification is a way to mitigate negative production as well as climate change impacts (Nendel *et al*., 2023). Recent examples of soybean extension to northern production regions include Europe (Central, Northern Europe), Far-East Russia (Amur, Primorsky regions), North-East China (Heilongjiang, Jilin), and Canada (Manitoba, Quebec, Saskatchewan).

## More than just maturity groups and *E*-genes

Soybean cultivars are commonly classified into 13 maturity groups (MGs) which provides a rough approximation with respect to their environmental adaptation. Moreover, a series of *E*-genes and the *J* locus have been described to control flowering time; most of them are orthologs of known Arabidopsis flowering genes (Cao *et al*., 2017; Dietz *et al*., 2022). Among all *E* loci described, *E1*, *E2*, *E3*, and *E4* are considered to have the strongest effect on flowering and maturity in high latitude environments (Kurasch *et al*., 2017). In the present issue, Zhu *et al*. (2023) take a new look at that matter, identifying important factors to facilitate breeding progress in adaptation of soybean to such higher latitudes. In a diversity panel of >1500 early maturity soybean lines subject to genome-wide association mapping, they describe 30 putative QTLs for flowering time and 27 QTLs for time to maturity. Among the candidate genes predicted from individual QTLs, the well-known *E* loci and the *J* locus were identified. The effect of *J* was previously known for adaptation to short day length regions (tropics) through down-regulation of transcription of the *E1* promoter (Lu *et al*., 2017); *J* haplotypes found here apparently affect high latitude adaptation as well, probably through interaction with the *E1* locus. In addition, the *Dt2* locus controlling semi-determinate stem growth was predicted as a candidate gene for affecting time to maturity. A new allele at the *E4* locus (*e4-par*) causing earlier maturity than other recessive alleles was found in soybean lines of mainly Central European origin (Zhu *et al*., 2023). The complexity of the regulatory network for controlling flowering and maturity time is further illustrated by the fact that individual *E*-haplotypes derived from allelic variants at loci *E1–E4* exhibit a wide range of maturity times spanning from MG 000 to MG I; this indicates a significant influence of additional factors (QTLs) on flowering time as also described for other sets of soybeans (Fu *et al*., 2017; Zhang *et al*., 2017; Liu *et al*., 2021). These additional QTLs were shown to exhibit a strong environmental interaction in Zhu *et al*. (2023), thus implicating a potential of the candidate genes (*GmAP1d* and *GmFRL1*) in breeding for going north.

## Fine-tuning plant architecture

Moving soybean production from drought-affected areas of South Europe to higher latitudes of Central and Northern Europe (Nendel *et al*., 2023) requires phenological adaptation and tuning of plant architecture for optimum performance in any particular environment. As shown by Zhu *et al*. (2023), this is facilitated by appropriate timing through flowering and maturity QTLs. Remarkably, they also identified the *Dt2* locus as a candidate gene for time to maturity in their diversity panel. Although the direct influence of *Dt2* on time to maturity appears to be small, particular combinations of flowering/maturity and *Dt2* alleles can produce highly distinct phenotypes suitable for various specific environmental needs (Box 1). In soybeans of late maturity backgrounds grown in lower latitudes, determinate genotypes (*dt1*) are utilized to avoid excessive plant height associated with lodging and to achieve timely maturity. In addition, tall determinate (*dt1-t*) alleles have previously been introduced as well and, in combination with appropriate flowering/maturity alleles at *E1–E3* loci, these combinations can also produce plant architectures with plant height and number of nodes adapted to high yielding environments (Kim *et al*., 2022). In contrast to late maturity genotypes, the semi-determinate (*Dt2*) stem growth character is more often utilized in early maturity soybeans (MG III to 000), as indicated by the rather frequent occurrence of the *Dt2* allele in the re-sequenced panel published here (Zhu *et al*., 2023). Moreover, significant proportions of recently released European commercial cultivars (LIDEA, 2022) and cultivars from the northern eco-region of China (Liu *et al*., 2015) carry the Dt2 trait which illustrates its relevance in early maturity soybean production. The *Dt2* locus is known to be in epistatic interaction with *Dt1*, and the dominant *Dt2* allele causing the semi-determinate stem growth habit has been characterized as a recent gain-of-function mutation that must have occurred after domestication, as it is not known from wild types of soybean (Ping *et al*., 2014). Detailed characterization of the Dt2 MADS box transcription factor has revealed multiple genomic binding sites and pleiotropic interactions with genomic regions modulating flowering time, water use efficiency, stomatal development, stress response, and hormone signaling (Zhang *et al*., 2019) which is prompting further investigation of agronomic traits affected to avoid possible trade-offs. Existing natural variation of *Dt2* was shown to also impact adaptation and yield-related traits (Liang *et al*., 2022).

**Box 1. Soybean plant architecture is controlled by stem termination and flowering time**

**Fig. 1.**
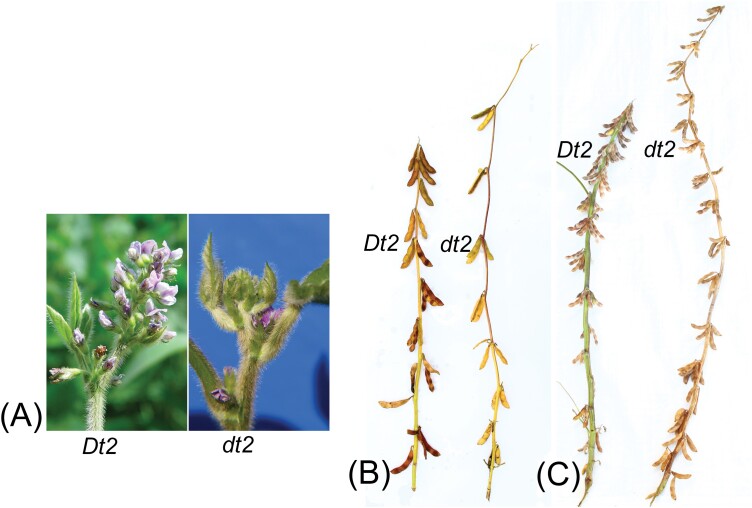
Soybean plant architecture and maturity time. (A) Terminal flower formation in the dominant *Dt2* genotype (left) and flower formation in stem nodes of the recessive *dt2* allele with indeterminate stem growth (right). (B) Early and (C) late maturity caused by different alleles at the *E1* and *E2* loci.

A MADS-box transcription factor is modulating semi-determinate versus indeterminate soybean stem termination (locus *Dt2* in genotypes with *Dt1* background). The dominant allele *Dt2* causes the cessation of stem elongation through the formation of a terminal flower raceme, whereas *dt2* causes indeterminate stem growth with flower formation in stem nodes only (Fig. 1A). Flowering and maturity time differences caused by different alleles at the *E1* and *E2* loci reveal either a very early (Fig. 1B; MG 000) or a later maturity (Fig. 1C; MG 0 to I). Different allelic combinations of *E* loci and *Dt2* (semi-determinate) versus *dt2* (indeterminate) stem termination have drastic effects on phenotype, with differences in stem length, stem diameter, number of nodes per stem, distribution of inflorescences and pods along the stem, and finally seed yield.

## Domestication, adaptation, and selection

Along with pod shattering, seed hardness, and several other characteristics, stem growth habit and flowering time are considered key domestication and diversification traits of soybean, and photoperiodic response genes such as *E1–E4* are playing a major role in local adaptation (Sedivy *et al*., 2017). With respect to earlier flowering date, selection processes from wild annual soybeans through farmers’ landraces and finally modern released cultivars, and from southern to northern genotypes have been described in the Chinese soybean germplasm pool in terms of allele changes at QTL regions (Liu *et al*., 2021). Both exclusion of alleles and emergence of new alleles occurred during these natural and artificial selection processes. The strongest reduction of wild alleles was found between landraces and released cultivars, and wild alleles were also reduced in the adaptation process from southern to northern populations. On the level of soybean *E*-genes, such processes of change in the occurrence of different allele frequencies and *E*-haplotypes between Asian, North American, and European populations were also described (Langewisch *et al.*, 2017; Miladinović *et al.,* 2018), reflecting adaptation processes to growing environments. Based on the QTL–allele matrix for time to flowering of the Chinese soybean germplasm pool analyzed, a further genomic selection potential for earliness of 4–11 d was predicted (Liu *et al*., 2021), which would additionally support the selection for adaptation to higher latitudes.

Finally, modulation of flowering time might also be important for changing soybean cropping patterns in agronomic adaptation to climate change for different production environments. This could involve either earlier or later sowing dates to escape periods of drought or high temperature during critical stages of development. Both strategies would require additional plasticity in adaptation to modified photoperiods, time to maturity, and duration of the seed filling period, as supported by the present findings (Zhu *et al*., 2023).
